# 3D integral imaging of acoustically trapped objects

**DOI:** 10.1038/s41598-023-50662-8

**Published:** 2024-01-02

**Authors:** Kooshan Mohsenvand, Artur Carnicer, Benedetta Marmiroli, Ali-Reza Moradi

**Affiliations:** 1 Department of Physics, Institute for Advanced Studies in Basic Sciences (IASBS), Zanjan, 45137-66731 Iran; 2 Departament de Física Aplicada, Universitat de Barcelona (UB), 08028 Barcelona, Spain; 3 Institute of Inorganic Chemistry, Graz University of Technology, 8010 Graz, Austria; 4 School of Nano Science, Institute for Research in Fundamental Sciences (IPM), Tehran, 19395-5531 Iran

**Keywords:** Applied optics, Optical techniques, Applied physics, Techniques and instrumentation

## Abstract

3D imaging provides crucial details about the objects and scenes that may not be obtained via 2D imaging methods. However, there are several applications in which the object to be 3D-imaged requires to be immobilized. The integrated digital holographic microscopy (DHM) and optical trapping (OT) system is a useful solution for such a task, but both DHM and OT are mostly suitable for microscopic specimens. Here, for the first time to the best of our knowledge and as an analogy to the DHM-OT system, we introduce integral imaging (InIm) and acoustic trapping (AT) integrated system for 3D imaging of immobilized mesoscopic and macroscopic objects. Post-processing of InIm data enables reconstructing the scene at any arbitrary plane, therefore, it re-focuses any particular depth of the object, which is a curtail task, especially when the object is trapped by AT. We demonstrate the capability of our system by simultaneous trapping and 3D imaging of single and multiple irregularly shaped objects with mm sizes.

## Introduction

3D imaging and 3D microscopy provide valuable structural and phenomenological details about the samples in various fields of science and technologies, such as the biology of living matter, physical characterization of materials, and morphometric investigations in surface sciences^1^. Therefore, especially in the past three decades, substantial efforts have been devoted to extend the 2D imaging modalities into their 3D analogs^2^.

Several approaches to this end have a scanning nature, and they use either light-matter interaction information or other types of interactions to map the 3D profile of samples. For example, in confocal microscopy, by the proper exclusion of the light from the specimen that is not from the microscope’s focal plane, not only better observation of details can be achieved, but also it is possible to build 3D reconstructions of a volume of the specimen by assembling a stack of thin slices taken along the vertical axis^3^, or in atomic force microscopy, the atomic forces between a probe tip and the outer surface of a sample map the 3D topography of the sample surface^4^.

However, several important applications require single exposure imaging, for which the aforementioned methodologies fail. An effective label-free and non-destructive technique, which can be performed in arbitrary time scales, from milliseconds to hours, and at video-rates is digital holographic microscopy (DHM)^5,6^. According to the interferometric nature of DHM it is very sensitive to mechanical vibrations and several attempts have been reported to overcome or reduce the vibrations^7^.

The limit on the sample size up to tens of micrometers and the requirement to use an illumination source with a high coherence degree, which is associated with the presence of speckle noises, demands a complementary 3D imaging approach. Indeed, a classification for the optical imaging techniques is based on the illumination source properties; being coherent or incoherent. It seems, therefore, a complementary 3D imaging approach that uses an incoherent light source and works for millimetric and beyond sized objects is demanded. Integral imaging (InIm) is an elegant candidate for it^8^. InIm is based on acquiring the multi-view directional information of a 3D scene. A micro-lenslet array (MLA) generates a set of 2D elemental images (EIs) on an imaging sensor^2^. The scenes captured in EIs have different perspectives according to the off-axial lateral positions of the lenslets of the MLA. In the reconstruction process of InIm, the 3D image of the object is numerically reconstructed by means of ray or wave optics methods and the volumetric information of it is extracted. In the display stage, the EIs are projected through a virtual pinhole array at arbitrary longitudinal distances in a computer^9^.

It is obvious that for many cases the DHM and InIm techniques should be applied when the sample to be imaged and analyzed is immobilized. In the case of DHM, there is a large class of objects of micrometer sizes, in particular living cells and aerosol particles, which need to be in a liquid or gaseous environment for their normal functionality^10^. Brownian motion and motility (for some living cells) and fluxes in the medium avoid the long time DHM imaging and tracking of samples without combining the DHM system with an immobilization technique. Using a pipette or other known physical or chemical methods for living cell immobilization on planar surfaces may induce undesirable effects in biosamples^11^. In addition, surface proximity effects become more pronounced when the size of the object decreases^10^.

One smart approach for immobilization of micron sized samples in suspension is using an optical trap (OT) or optical tweezers^12^. The method was invented and developed by Artur Ashkin and uses the radiation pressure of a laser beam that is focused through a high numerical aperture microscope objective^13,14^. With OTs, samples can be kept and studied for up to several hours. Moreover, multiple OTs have been used for controlled manipulation of single cells to achieve tomographic phase microscopy image or 3D refractive index map of cells and bacteria and even detecting intracellular changes^15–18^. OT, up to a high extent, is nondestructive and non-invasive, which makes the technique very useful for a variety of biological and medical studies including cell living processes^19^. Therefore, it is extensively used for many applications, such as applying and measuring forces in the pN range, confining dielectric micro-spheres, bacteria, and living cells^20^.

Given the aforementioned features of OT and DHM, it seems both are capable of being integrated. Since both DHM and OT techniques can be inserted in a conventional microscopy setup, the combination of them is straightforward. Multiple configurations for the combination of the methods can be considered and used for 3D quantitative visualization of 3D structures that are trapped by the laser beam^14,21–23^.

For bigger size objects, which are subjected to InIm, however, OT cannot be the proper immobilization technique. Forces inserted by OT can dislodge or trap objects of up to tens of microns, therefore, for trapping and manipulation of bigger sizes other methodologies should be considered. On the other hand, there are several important mm sized objects that require to be immobilized while they are subjected to imaging and studying. Among living cells, Zebrafish, Egg cells, and Amoeba Proteus are in such ranges, to name a few^24,25^. Further, several lithographically fabricated samples in such ranges may require to be confined and 3D imaged^26^. For example, small-sized gears and turbines that are fabricated for studies in the field of soft matter or statistical mechanics problems need to be 3D imaged and for that aim they should be kept immobilized^27^.

The proper alternative to OT for the immobilization of objects in such sizes is acoustic trapping (AT)^28^. Acoustic waves can exert non-contact forces on rather bigger sized objects, and the formation of trap sites using such waves provides a complementary technique to OT, which is called acoustic trapping (AT). At the points that these forces converge, the levitation of particles of a wide range of materials and sizes through the air, water or biological tissues becomes possible^29^. Therefore, AT is very significant for several applications, such as particle studies, cell-cell interactions, cell population, size sorting, etc.^29–32^. The arrangement of single-axis levitators which consist of an ultrasound transducer (UT) and a reflector or another UT above can be used for generating AT by considering the standing acoustic waves. Standing waves include several nodes and anti-nodes, and nodes have the potential that is necessary for trapping particles. Further, by varying the phase difference between the UTs, the traps can be moved without mechanical actuation. Various configurations for 2D and 3D manipulation of particles with AT have been considered^31^. However, the controlled 3D trapping, translation and rotation with a single-sided array UTs is a more straight counterpart of OT at the larger-scales.

In this research, we show that, similar to the integration of DHM and OT, the integration of InIm and AT is possible. The two methods are synergistic, and their combination is possible. Post-processing of InIm data enables reconstructing the scene at any arbitrary plane, therefore, it re-focuses any particular depth of the object, which is a curtail task, especially when the object is trapped by AT. Both methods have specific applications in a wide range. However, the nature of the InIm method that uses incoherent light, the easy implementation of AT, and the wide range of usable object size, make the integration of the methods synergistic and suitable for several important applications. We consider a simple arrangement to prove the concept and perform validating experiments along with discussing the possibilities of the combination configurations.

## Theory

### Acoustic trapping

The acoustic wave is mechanical and longitudinal, and the superposition of two acoustic waves forms a standing wave. In standing waves, the mechanical wave creates acoustic pressures along the propagating direction, therefore, any standing wave includes a number of nodes and anti-nodes, and the nodal points may be used for AT^29^. However, there are several ways to produce an AT; for example, a single acoustic wave incident on a rigid wall, by superposition of the initial wavefront and the back-scattered one can also form a standing wave and an AT, in turn. In this research, we use counter-propagating acoustic waves by adjusting sets of acoustic source arrays in front of each other in a vertically arranged setup.

The theoretical description of acoustic radiation forces, similar to the optical counterpart, is performed by considering the scattering and absorption of acoustic waves. However, in the acoustic case the forces are derived from the dynamic Navier-Stokes equations for fluid mechanics^29^. The total acoustic force acting on a particle along the axial direction can be expressed as:

1 $$\begin{aligned} {\textbf{F}}_{\mathrm{{ac}}} = {\textbf{F}}_{\mathrm{{grad}}} + {{\textbf{F}}_{\mathrm{{scat}}}} + {{\textbf{F}}_{\mathrm{{abs}}}} + {{\textbf{F}}_{\mathrm{{str}}}}, \end{aligned}$$

where ${\textbf{F}}_\mathrm{{grad}}$, ${\textbf{F}}_\mathrm{{scat}}$, ${\textbf{F}}_\mathrm{{abs}}$, and ${\textbf{F}}_\mathrm{{str}}$ represent trapping, scattering, absorption and streaming components. By the radiation forces derived from the Navier-Stokes equations, the trapping and scattering forces ($\textbf{F}_{\mathrm{{grad}}}$ and $\textbf{F}_{\mathrm{{scat}}}$) can be described. Moreover, the acoustic streaming effect ($\textbf{F}_{\mathrm{{str}}}$), which is caused by momentum transferring from the flowing surrounding medium to the object in the AT, can be derived. It is also possible that part of the acoustic radiation is absorbed by the particle in the AT which is expressed as ${\textbf{F}}_\mathrm{{abs}}$. The formulae for the terms in Eq. 1 can be derived by perturbation analysis^33^. In such analyses, the harmonically oscillating standing pressure and velocity in sound waves average to zero, and the lowest non-vanishing order of the time-averaged fields, i.e., pressure (*p*), density ($\rho$), and velocity (*v*), which contribute to the acoustic forces are second order perturbations.

In many practical cases, especially in biomedical applications, among the different terms in Eq. 1, it can be shown that the gradient force is more effective than other forces and other parts can be neglected^33^. Depending on the predesigned arrangements of the acoustic sources and properties of the object in the nodal area, the gradient force can trap the object toward the nodal point. By smart arrangements of the acoustic source arrays one can superpose the gradient forces to achieve a tight AT^28,29,33,34^.

The perturbation analysis to calculate the gradient force in the Rayleigh regime, where the size of the particle is considerably smaller than the acoustic wavelength in the fluid, leads to the Gor’kov potential theory. The Gor’kov potential, *U*, is the overall potential energy in a nodal point and the gradient force can be written as^35^:

2 $$\begin{aligned} \textbf{F}_{\mathrm{{grad}}} = {-\nabla U}. \end{aligned}$$

Although Gor’kov potential assumes a friction-free fluid and spherical fluid particles, yet, his potential theory has shown to be applicable to several acoustofluidics systems^29^. The Gor’kov potential is a function of the average acoustic pressure, average wave velocity, the size of the sphere in the AT, the compressibility and density of the fluid in which the standing waves are formed, and the material dependent factors^33^:

3 $$\begin{aligned} U=\frac{4{\pi }{a^3}}{3} {\Big [}f_1\frac{1}{2}\kappa _0\langle p^2 \rangle -f_2\frac{3}{4}\rho _0\langle v^2 \rangle {\Big ]}. \end{aligned}$$

In Eq. 3, ${\langle }{p^2}{\rangle }$ and ${{\langle }{v^2}{\rangle }}$ are the time-averaged acoustic pressure and velocity, respectively, *a* is the particle size, and $\kappa _0$ and $\rho _0$ are the compressibility and density of the host fluid, respectively. The material-dependent pre-factors are given by $f_1=1-\frac{\kappa _p}{\kappa _0}$ and $f_2=\frac{2(\rho _p-\rho _0)}{(2\rho _p+\rho _0)}$, where the subscript *P* denotes a property of the particle. In Eq. 3, the first and second terms correspond to contributions of a monopole due to radial oscillations and a dipole due to linear oscillations of the particle, respectively. The patterning of the particles inside the acoustic pressure landscape depends on $f_1$ and $f_2$ which are the acoustic contrast parameters of the particle and the surrounding fluid, respectively. For the very simplest form of the acoustic field, a 1D sinusoidal standing wave of sound pressure $p={p_0}{\cos (kx)}{\sin ({\omega }t})$, where $\omega$ is the angular frequency, *x* is the position and *t* is time, Eq. 2 can be simplified to

4 $$\begin{aligned} |\textbf{F}_{\mathrm{{grad}}}|=-4{\pi }{\phi }kE{a^3}{\sin (2kx)}, \end{aligned}$$

where $E =\frac{p^2}{4{{\rho }_0}{c^2_0}}$ is the acoustic energy density, $c_0$ is the speed of sound in the host medium, ${\phi }=\frac{1}{3}{f_1}+{\frac{1}{2}{f_2}}$ is often referred to as the acoustic contrast factor and $k=\frac{2\pi }{\lambda }$ is the acoustic wavenumber in the host fluid^29^.

### Integral imaging

InIm is based on a simple idea presented by Gabriel Lippmann^36^, and it is inspired by the mechanism of the human visual system. Human visual systems include two eyes that are separated at a distance, which makes a perspective between pictures of each eye. The acquired images along with the adaptation mechanism of the eye and the complex processing of the brain enable humans to see 3D scenes. In InIm, a similar perspective is obtained by incorporating an MLA or, alternatively, through a moving camera, which provides multiple elemental 2D images at different viewing angles. The ability of InIm to reconstruct a particular plane in different depths, helps us finding depth information for multiple objects using a single InIm data.

**Figure 1:**
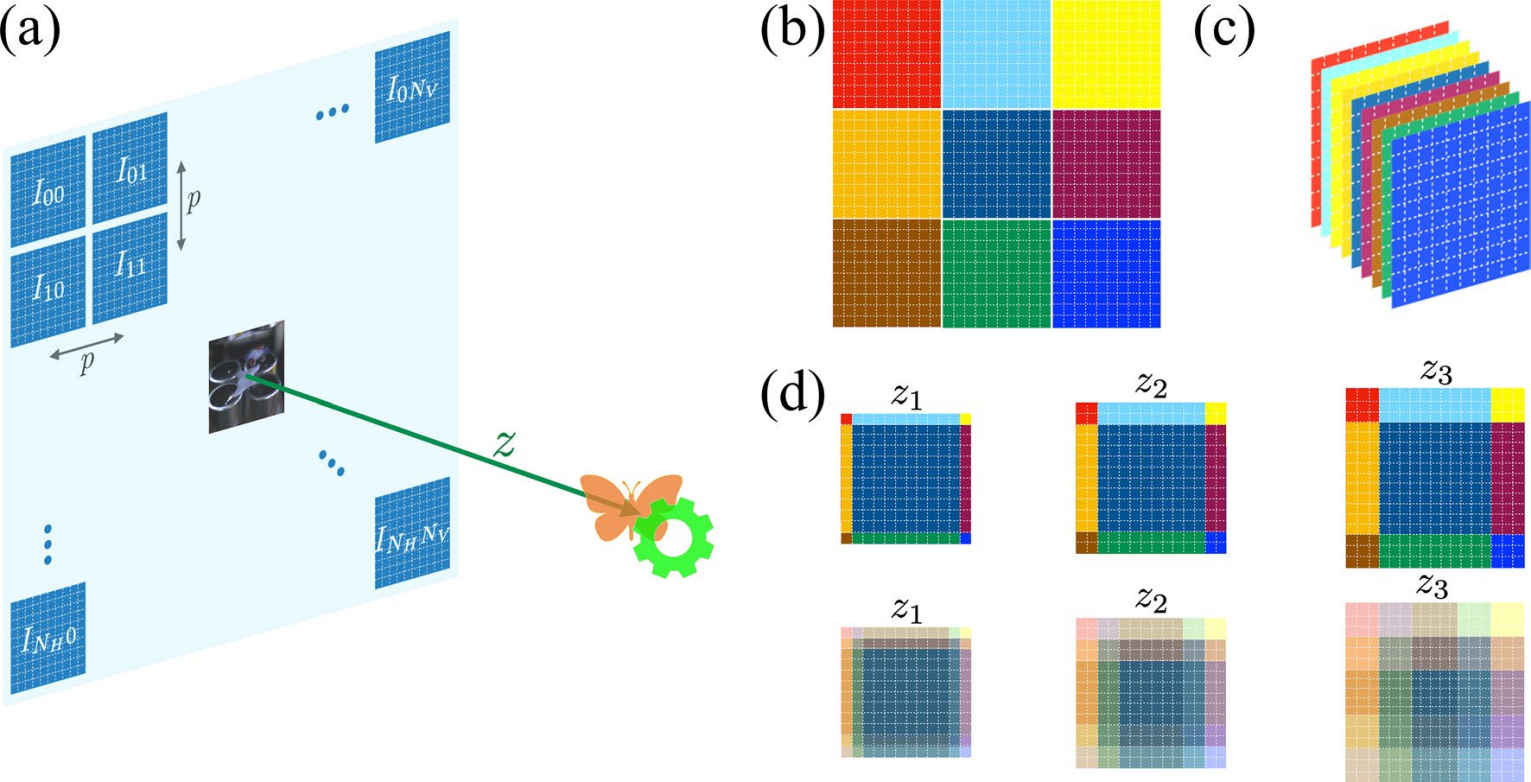
(**a**) Sketch of the SAInIm recording approach: *p* is the distance between EIs, $I_{k,l}$ is the $k,l-$EI and *z* is the pick-up distance. $N_H$ and $N_V$ are the number of acquired EIs in horizontal and vertical directions, respectively. (**b**) Scheme of a 3 $\times$ 3 EIs set. (**c**) Sum of the EIs, which represents the zero refocusing. (**d**) The refocusing procedure to three different planes of $z_1$, $z_2$ and $z_3$. The lower row shows the associated changes during the focusing for each pixel (see Fig. 17^2^).

As explained above, the 2D EI set of a 3D scene is recorded by an MLA and a fixed camera in a single shot, or alternatively by a scanning camera and its single lens, while the focus of the imaging system is adjusted to the middle depth of the scene. In the case of MLA the area of the sensor is shared among all the views, which means that they are recorded in low resolution. This drawback can be overcome by using the scanning camera and following the so-called synthetic aperture integral imaging (SA-InIm) strategy: The camera is displaced across the recording plane and every EI is recorded in its corresponding position; in this way, all EI take advantage of the full sensor. Nevertheless, the method requires taking as many snapshots as EIs and thus, it cannot be used with fast dynamic scenes. Since in this work we deal with immobilized samples, the SA-InIm approach is an appropriate choice. As shown in Fig. 1a the EIs are arranged as $I_{00}$, $I_{01}$, etc. In our experiments, in the horizontal and vertical directions we acquire $N_H$ = 10 and $N_V$=10 EIs, respectively.

The reconstruction procedure is performed by the “shift and sum algorithm”^2,9^, which is schematically shown in Fig. 1. In order to simplify the explanation on the image reconstruction process, we consider an array of 3 $\times$ 3 EIs, and each EI is shown with a different color for easy tracking (Fig. 1a). The extension to bigger size arrays is straightforward. First, as shown in Fig. 1c, the recorded EIs are overlapped and lead to a matrix with the same size of an EI and in $N_H \times N_V$ layers (9 in our case). Then, following the pattern of Fig. 1a each EI is shifted for one pixel toward its original position on the camera sensor for the first plane of reconstruction ($z_1$). For the second plane ($z_2$) the shift is applied for two pixels and so on. Figure 1c is the sum of the EIs, i.e., the zero refocusing without any shift, and Fig. 1d shows the refocusing procedure to three different planes of $z_1$, $z_2$ and $z_3$. The lower row of Fig. 1 shows the associated changes during the focusing for each pixel. These operations are carried out repeatedly along the axial axis to achieve a volumetric reconstruction of a 3D object, i.e. *I*(*x*, *y*, *z*) at the plane *z* (pick-up distance). The “shift and sum equation” summarizes the operations as:

$$\begin{aligned} I(x,y,z) = \sum _{k}^{N_H}\sum _{l}^{N_V}{I_{k,l}\left( x-k\frac{N_x p f}{c_x z}, y-k\frac{N_y p f}{c_y z}\right) }, \end{aligned}$$

where $I_{k,l}$ describes the $k,l-$ EI, and $N_x \times N_y$ and $c_x \times c_y$ are the number of pixels and the size of the camera sensor, respectively. *f* is the focal distance of the camera objective and *p* is the distance between EIs in horizontal or vertical directions. It is worth pointing out that the number of pixels $N_x \times N_y$ limits the number of planes *z* where *I*(*x*, *y*, *z*) can be reconstructed.

## Results and discussion

**Figure 2:**
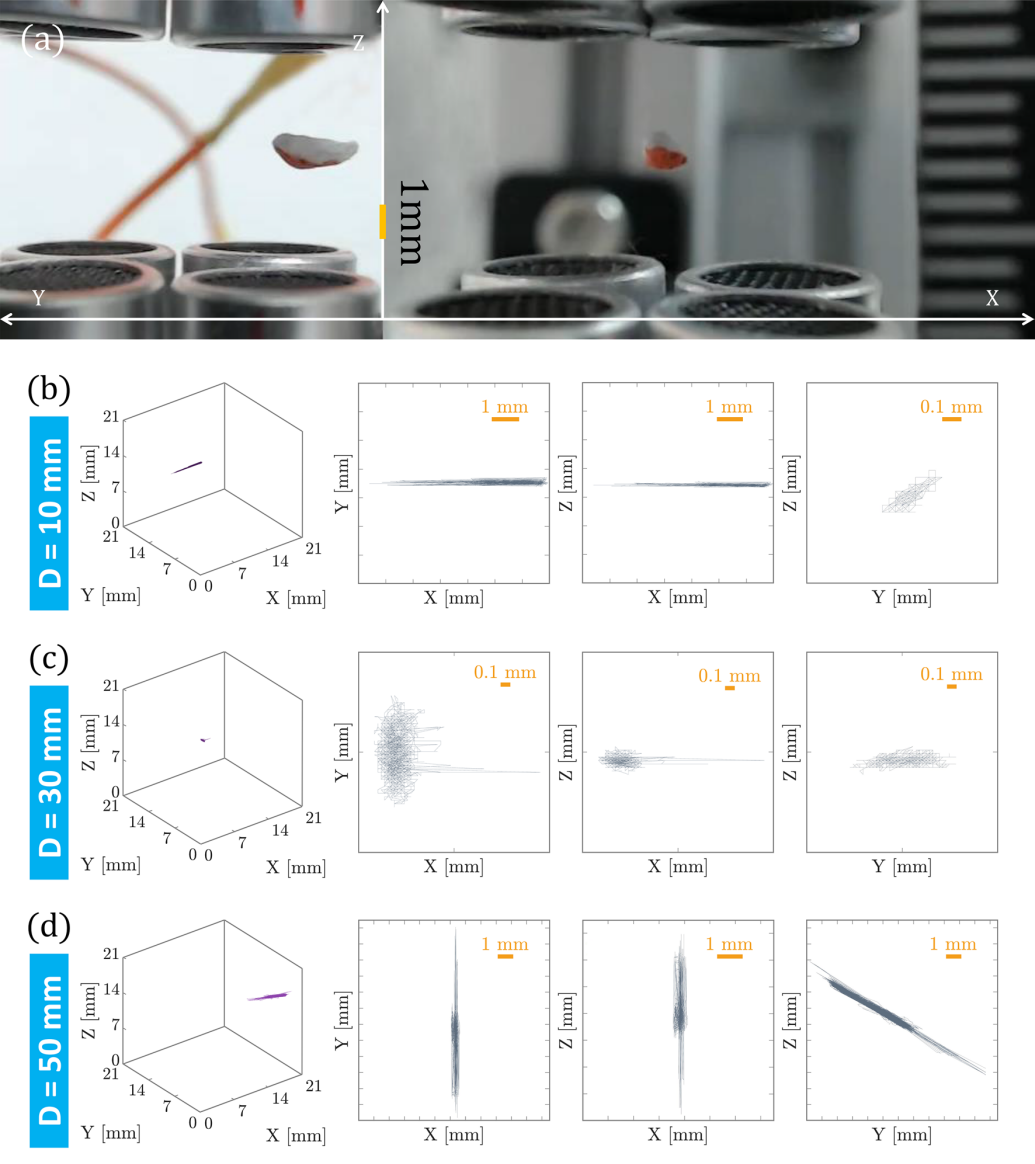
Trajectory analysis of the motion of an object in the AT. (**a**) The orthogonally positioned and synchronized cameras capture images at the 25 fps rate. 3D trajectories and 2D projection of the trajectories for the distances D = 10 mm, D = 30 mm, and D = 50 mm, between the upper and lower UTs are shown in panels (**b**), (**c**), and (**d**), respectively. The trajectories show that there is an optimized distance between the UT arrays, in which the objects are trapped in 3D more stably.

Similar to OT systems, the calibration of the trap stiffness can be useful for knowing the setup capability to hold and manipulate the trapped objects^10,34^. To perform such a task, we provide a trajectory-based investigation on the stiffness of the ATs. The procedure is demonstrated for a simple arrangement of the UT arrays used in our experiments. Using a couple of identical synchronized cameras (Logitech, C922, 25 fps) and positioning them orthogonally, the images of a trapped particle in XZ and YZ planes are captured within time. The trapped particle has a size of approximately 1 mm$\times$ 1 mm $\times$ 2 mm and its projected images in XZ and YZ planes in time provide the 3D trajectory.

Figure 2a shows the typical images of a trapped object captured by the aforementioned orthogonal cameras. The successively captured images are subjected to center-of-mass based tracking procedures and the 3D position of the trapped object within time is extracted. In this process, the captured images are cropped and converted into gray scales. Then, their binary images are obtained by defining a threshold, and the center of the bright area is taken as the position of the particle. The procedure is performed for the image sequences of both XZ and YZ cameras to obtain (*x*(*t*), *y*(*t*), *z*(*t*) information about the trapped objects. The stability of the trap depends on the distance D between the counter-propagating UT arrays. We examine the stability of the trap for several distances. In Fig. 2b–d the corresponding 3D trajectories and 2D projections of the trajectories for the distances D=10 mm, D=30 mm, and D=50 mm are demonstrated, respectively. The examination is performed for more than 5 min and the images are acquired at the rate of 25 fps. Each condition is tested at least three times. The trajectories in Fig. 2 clearly show that there is an optimized distance between the upper and lower arrays of UTs, in which the objects are trapped in 3D by AT more stably. However, if trapping in some specific direction or plane is of concern, the results indicate that other distances can be considered. For example, when D=10 mm, the particle is stably trapped in the Y-Z plane, even if it has almost freely moved along the X direction. Such cases can be useful for applications, in which the collection of small particles requires staying freely in a plane without utilizing a mechanical mount. The different behavior in the X and Y directions, while the system theoretically is symmetric in those directions, is attributed to the possible misalignment of the UTs.

To validate the present methodology on InIm-AT integration, we trapped two millimeter sized objects by AT and applied the InIm technique. The trapped objects are made of polystyrene in irregular shapes (a butterfly and a gear).

**Figure 3:**
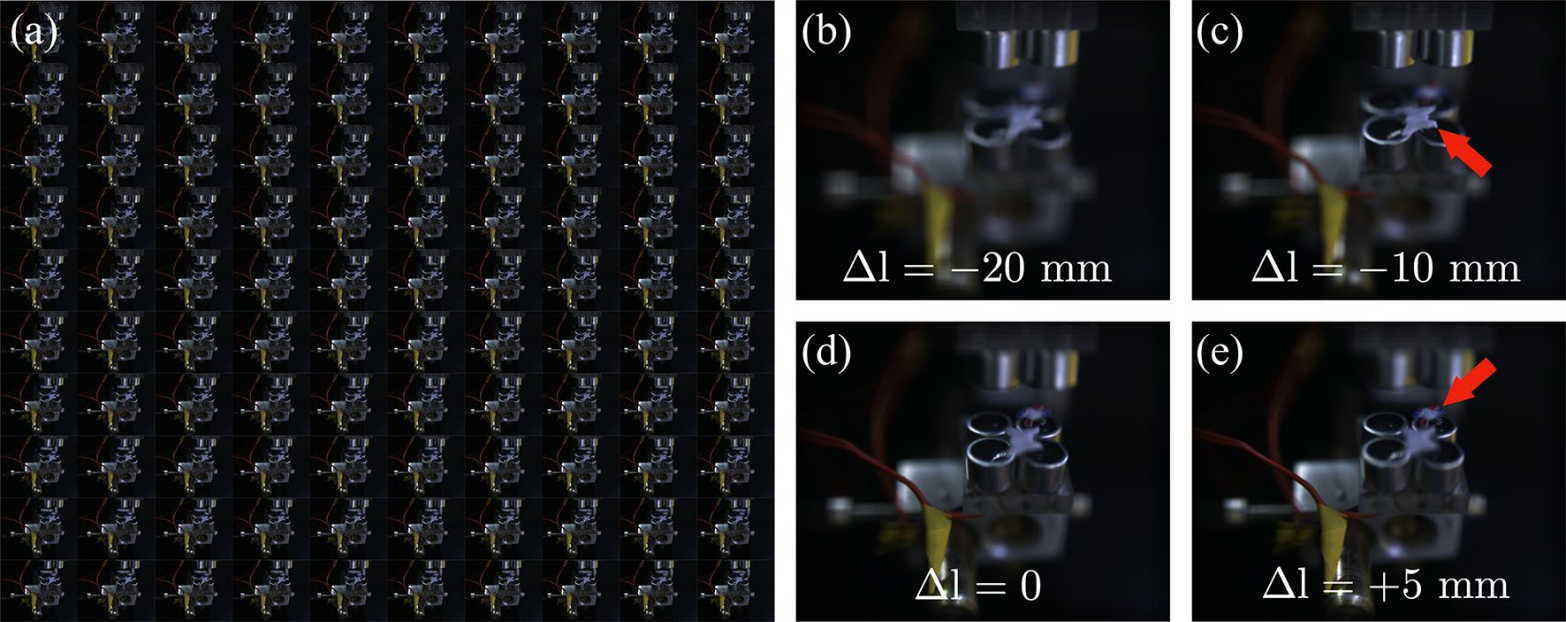
(**a**) The EIs of a butterfly and a gear shape objects immobilized by AT apparatus, and the refocused images obtained by InIm reconstruction at (**a**) $\Delta l = -20$ mm, (**b**) $\Delta l = -10$ mm, (**c**) $\Delta l$ = 0 and (**d**) $\Delta l = +5$ mm. In $l = 90$ and *l* = 105 mm, the butterfly and the gear shape objects are in focus. Therefore, their distance is computed as 15 mm, which is in agreement with their actual distance. The full refocusing is shown in the Supplementary video S1. The objects are marked with a color marker for detection purposes.

In our demonstration experiment, we acquired 100 2D images. The acquisition of the set of EIs can be performed by the use of MLA, in which according to the single shot nature the presence of ambient vibrations and other mechanical, thermal or optical noises will not influence the 3D imaging. However, as we mentioned earlier the use of MLA limits the number of pixels and requires a huge active area of the recoding device. The method of scanning camera although provides high-resolution elemental images, requires as many shots as the number of elemental images. The acquisition time depends on the speed of the scanning device, which can be very high if advanced scanning devices are used. The 100 images of Fig. 3a took 10 min. The range of particle vibrations inside the acoustic trap is less than 1 mm, which given the size of the imaging scenes, the size of the images and the pixels, and the refocusing steps in the reconstruction process, the vibration can be safely ignored. Therefore, there is a compromise between living with such noises in the trap and the resolution of the final reconstructed images, considering the dynamics of processes or phenomena one can choose to either use MLA or a scanning camera. The EIs are subjected to the reconstruction process. The numerical reconstruction, which is also performed for the present research, provides 3D images, numerical focusing or quantitative depth information. The experimental procedure follows the synthetic-aperture integral imaging capture arrangement and the associated reconstruction is applied. The details of the procedure are described in^2^. In this arrangement, the camera initially is focused on the object at a distance of $l=$100 mm, with a wide-angle lens of ${f}^{\#}=1$. We captured a set of $N_H=N_V=10$ EIs with a pitch of $P_H=P_V=1$ mm. The sensor size is 6.656 mm $\times 5.325$ mm with 1280$\times$1024 pixels. The size of the sensor pixel in *x* and *y* is $\mu$m. Figure 3a shows the captured EIs. The InIm reconstruction enables post-refocusing of the irregular objects in the scene into arbitrary depths. The difference of the distance of a specific depth from the initial camera distance is indicated by $\Delta l$. As shown in Fig. 3, in panels (c) and (e) the butterfly and the gear shape objects are in focus and seem sharp at $\Delta l = -10$ mm and $\Delta l = +5$ mm, respectively. This, consequently, determines the reconstructed distance between the two objects, which is 15 mm and is in full agreement with their actual distance. The full refocusing procedure is shown in Supplementary video S1.

**Figure 4:**
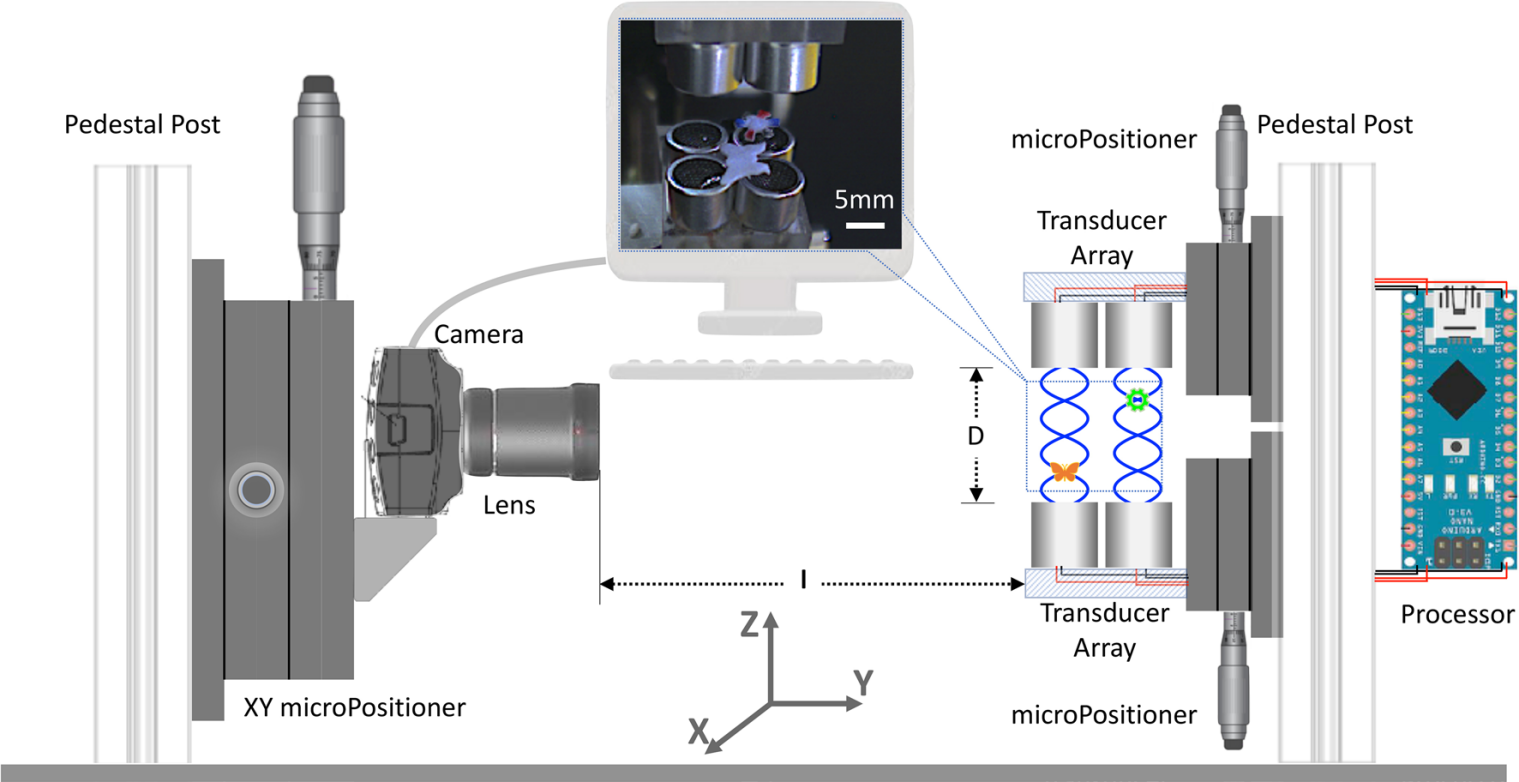
Scheme of the AT-InIm experimental setup. It is used to trap two irregular objects by AT apparatus consisting of 8 UTs while the distance between the upper and lower UTs varies. InIm part, which is placed at *l* = 100 mm away from the AT apparatus, includes an imaging camera, a lens, and an XZ micro-positioner to scan the sample and resemble a microlens array. Inset: a photo of the multiple AT trapped objects acquired by the camera equipped with a wide-angle lens.

## Conclusion

In conclusion, we combined acoustic trapping and integral imaging methods for 3D imaging of to-be-immobilized objects of sizes that cannot be trapped by optical trapping. We characterized the acoustic traps by analyzing the 3D trajectory of an irregularly shaped trapped object, toward determining the conditions for an optimized trapping experiment. Post-processing of integral imaging data enables reconstructing and re-focusing to any particular depth of the object in the acoustic trap. Due to this advantage, the integrated system may find several applications for multiple disciplines, including biomedicine and fluidics. Both methods have specific applications in a wide range. However, considering the nature of the integral imaging method that uses incoherent light, the easy implementation of acoustic tweezers, and the wide range of usable object size, the present system will find numerous important applications, and it may be potentially considered as a bench-top device. For example, the integrated system can be used to study the specimens, e.g., biological samples, for which the influence of surrounding walls of the containing chamber is a disturbing factor^37^. In particular, as AT is now considered also as a novel fabrication tool for microfabrication^38^, the coupling with InIm could improve the correct positioning and alignment of the elements which are not spherical to produce micro-arrays.

## Methods

### Experimental setup

Figure 4 shows the schematic of the InIm-AT setup. AT part includes a couple of 4 UT arrays (AUDIOWELL, SPU1040AOH07T/R, Housing material: Al, Capacitance: 2400 pF, Sound pressure level: 105 dB, Operating frequency: 40 kHz, Diameter: 10 mm). The two arrays are mounted on two horizontal platforms (up and down) and each platform can move up and down by a micropositioner, so it allows adjusting the distance D between the arrays in a range of 10–60 mm. The counter-propagating UTs perform standing waves. The UTs work at 40 kHz natural frequency, and their onset and adjustment are performed by a custom-made control system. This system adjusts several parameters of the generating waves, such as their shape, amplitude, and apparent frequency. This control system includes an Arduino Nano (ATmega168, Operating voltage: 5 V, Current per Pin: 40 mA, Clock speed: 16 MHz) as the central processor and a driver motor (L298N, Double H bridge drive, Logical voltage: 5V, Drive current: 2 A) as the conductor of the UT. Before data acquisition, the control system and the UTs performances are monitored and validated by an oscilloscope (GWINSTEK GOS-620 Oscilloscope, 20 MHz). For the InIm experiments, the imaging camera can be equipped with either an MLA or a 2D scanning apparatus. Another approach is using an array of digital cameras. Despite that, the EIs will have high resolution and lead to high parallax 3D images. However, the system will be very bulky, and precise synchronization of numerous digital cameras will be required. Moreover, the minimum pitch will be limited to the size of the cameras. The use of the SAInIm approach, is an elegant possibility, since the main advantage of a moveable camera over a multiple-camera system is its flexible pitch and parallax. A useful possibility of SAInIm approach is that it enables acquisition of the EIs within an arbitrary array, however, for convenience we use a square grid for recording the EI set.

However, the main drawback of the moving camera approach is the large acquisition time that avoids 3D imaging of dynamic phenomena. This can be overcome by scanning devices with scanning speeds more than the timescale of the sample dynamics. In our experiments, for a single InIm image we recorded 100 EIs by moving the color camera (DCC1645C, Thorlabs, 8 bit dynamic range, $\mu$m pixel pitch) in a 10 $\times$ 10 mesh of 1 mm inter-distance using a micro-pisitioner. A wide angle ($>70^{\circ }$) lens (LM6NCL, 6 mm) is mounted on the camera, so that all the trapped objects and the UTs can be viewed, as shown in the inset of Fig. 4. Obviously, the procedure can be conducted, instead, in a single step recording if a proper MLA is used. The aforementioned configuration for the InIm-AT system is the most straightforward combination. However, if somehow the effect of gravity is overcome, the AT apparatus may be mounted at any tilted direction regarding X or Y directions, while the InIm apparatus remains the same as in Fig. 4 The trapped and 3D objects are made of polystyrene and are punched with a punching machine in a butterfly shape and a gear shape with a maximum size of 1$\sim$7 mm and thickness of $\sim$1 mm. The objects are also marked with a color marker for detection purposes. Once the objects are brought into the center of the AT apparatus they re-align themselves within 1–2 s in order to find the maximum volume trapping region and their rotation is controlled considering the multiple cross-combining acoustic waves^39,40^.

We generate square waves to achieve trap sites, as compared to sinusoidal waves their generation and digital handling are more feasible. However, the transducers through their resonance behavior filter out almost all the frequencies and their output is a near sinusoidal wave. The peak-to-peak voltage ($V_{\mathrm{{pp}}}$) that the transducers receive is set to 13–15 V and the resolution of the phase difference between the outputs of the transducers is $\frac{\pi }{12}$. Based on the requirements of the device and the power required for the trapped particle, the voltage is readjusted, as the trapping power relates to the weight, shape, and density of the particle. In order to manipulate the AT trap sites, the electronics drive two channels. One channel is kept at a constant phase, while the phase of the other channel is shifted properly to move the trapped particles upwards or downwards^28^.

### Supplementary Information

Supplementary Video.

## Data Availability

Data underlying the results presented in this paper are not publicly available at this time but may be obtained from the authors upon reasonable request.
